# Radiation-Induced Emesis (RIE) in Extended-Field Radiotherapy for Gynecological Malignancies: Dosimetric and Non-Dosimetric Factors

**DOI:** 10.3390/curroncol28050308

**Published:** 2021-09-17

**Authors:** Yu-Ming Wang, Yi-Fan Chen, Pei-Yi Lee, Meng-Wei Ho, Eng-Yen Huang

**Affiliations:** 1 Department of Radiation Oncology, Kaohsiung Chang Gung Memorial Hospital, Chang Gung University College of Medicine, Kaohsiung 833, Taiwan; scorpion@cgmh.org.tw (Y.-M.W.); eivancubit@cgmh.org.tw (Y.-F.C.); pylee@cgmh.org.tw (P.-Y.L.); 2 School of Traditional Chinese Medicine, Chang Gung University, Kaohsiung 33302, Taiwan; 3 Department of Radiation Oncology, Xiamen Chang Gung Hospital, No. 123, Xiafei Rd., Haicang District, Xiamen 361126, China

**Keywords:** radiation, emesis, small bowel, dosimetry, extended-field radiotherapy

## Abstract

Radiation-induced emesis (RIE) is usually noted during abdominal-pelvic radiotherapy. In gynecological malignancies, it is usually noted in para-aortic but not whole-pelvic irradiation. Irradiated small bowel (SB) may be associated with RIE. The significance of SB dosimetry remains unclear. Dosimetric and non-dosimetric factors were evaluated and correlated with RIE in 45 patients with gynecological malignancies undergoing extended-field radiotherapy (EFRT) (median 45 Gy) from 2006 to 2021. Early-onset RIE (within 72 h after the first fraction of EFRT) was noted in 10 of 12 RIE patients. RIE was significantly associated with the SB mean dose. The RIE rates were 58.3% and 15.2% (*p* = 0.007) in patients with a low (<63%) and high (≥63%) SB mean dose. Logistic regression revealed that the SB mean dose remained the independent factor of overall RIE (*p* = 0.049) and early-onset RIE (*p* = 0.014). Therefore, constraint of the SB mean dose limited to less than 63% of the prescribed dose is suggested to decrease RIE.

## 1. Introduction

Radiation-induced emesis (RIE) is a common side effect in radiotherapy for abdominal malignancies. The incidence is around 40% [1]. Quality of life is always affected due to characteristic of early onset and the impairment of food intake [1]. The upper abdomen is a more frequent site than the pelvis for the development of RIE [2,3]. Therefore, the prevention and management of RIE can avoid treatment interruption of radiotherapy. Dosimetric study for RIE may be helpful for RIE prevention. However, there is no dosimetric study about RIE in patients with abdominal malignancies. The aim of the current study is to identify dosimetric factors of RIE in these patients.

## 2. Materials and Methods

### 2.1. Patients and Radiotherapy

From September 2006 to June 2021, 45 patients who underwent extended-field radiotherapy (EFRT) to treat the whole pelvis and para-aortic lymph node (PALN) for cervical or endometrial cancers were retrospectively reviewed. Patients who met the following conditions were eligible in this study: (i) cervical or endometrial cancer confirmed by histology; (ii) clinical FIGO stage IB2-IVa cervical cancer or pathologically stage IIIC cervical cancer or stage IIIC endometrial cancer; (iii) no prior radiotherapy; (iv) age ≥20 years old and performance status of the Eastern Cooperative Oncology Group (ECOG) 0–2; (v) adequate bone marrow, renal and liver function.

Axial CT slices were acquired every 3–5 mm with thermoplastic mask fixation, supine, and arm elevation position. Treatment planning was performed using the Pinnacle treatment planning system (Philips Radiation Oncology Systems, Fitchburg, WI) or RayStation treatment planning system (RaySearch Laboratories, Stockholm, Sweden). Organs at risk (OARs) such as the kidney, spinal cord, bladder, loops of small bowel, colon, and rectum were contoured. Contouring of the bowel was based on the following principles. The rectum was delineated first. Furthermore, the colon was contoured above the rectum to trace slice by slice from sigmoid to descending, transverse, and ascending colon. The remaining bowel loops were defined as small bowel. The constraint was kidney V20 < 30% for IMRT. Two-dimensional (AP/PA) or three-dimensional conformal radiotherapy (3D-CRT) using the 4-field technique, intensity-modulated radiotherapy (IMRT), or volumetric modulated arc therapy (VMAT) was delivered. Common Terminology Criteria for Adverse Events (CTCAE) version 3 was used for vomiting during the whole period of treatment recorded by a physician (E.-Y.H.) who was interested in radiation-induced bowel complications. In patients without symptoms, we routinely evaluated weekly. While patients had significant radiation-related side effects such as cramping, diarrhea or vomiting, immediate medications were prescribed for symptoms relief. The clinical target volume (CTV) delineation includes the vagina, external iliac, internal iliac, common iliac lymph, and para-aortic nodes for patients with hysterectomy. Intact uterine and cervix were additionally included in CTV for definitive radiotherapy for cervical cancer. In general, planning target volume (PTV) was an extension of CTV plus 10 mm in all directions. For patients who were treated with a daily image-guided setup, the PTV extension was 5 mm. The prescribed dose was 39.6–50.4 Gy/20–28 fractions for the whole pelvis and PALN. After EFRT, lymph node/parametrial boost and high-dose-rate brachytherapy were provided dependent on the condition of the disease. Further boost to 54~60 Gy to gross lymph node was delivered. The boost dose for an intact cervix was 24–27 Gy/4–6 fractions using brachytherapy. Cisplatin-based concurrent chemotherapy was provided for 39 patients. SB mean dose was presented as Gy or a percentage per fraction (%) with standardization to a 45 Gy prescription.

### 2.2. Statistics

The receiver operating characteristic (ROC) curve was used to determine the optimal cutoff SB volume and mean SB dose. Spearman’s correlation was used to calculate the association among BMI, SB volume and mean SB dose. An independent t test was used to compare dosimetry between two groups. A chi-square test was used for comparison of incidence of vomiting between different groups. Logistic regression was performed for prediction of vomiting. The statistics were proceeded by SPSS 25 (SPSS Inc., Chicago, IL, USA). 

## 3. Results

### 3.1. Dosimetric Data between Patients with and without RIE

There were 45 patients reviewed in this study. The characteristics of the patients are shown in Table 1. Grade 0, 1, and 2 vomiting were noted in 33 (73.3%), 7 (15.6%), and 5 (11.1%) patients, respectively. The onset of vomiting usually appeared within 72 h after the first fraction of radiotherapy in 10 patients. Two patients experienced RIE at 16 and 18 fractions after EFRT. Therefore, we defined early-onset RIE as an episode within 72 h.

**Figure 1:**
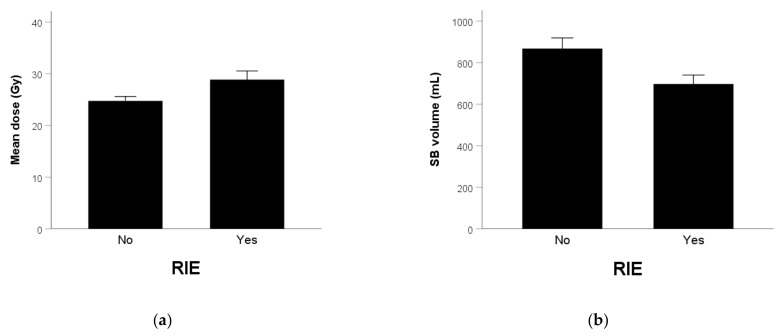
(**a**) Mean SB dose (*p* = 0.022) and (**b**) SB volume (*p* = 0.062) in patients without and with RIE.

The mean dose of SB was 28.9 ± 1.7 and 24.8 ± 0.8 Gy (Figure 1a) in patients with and without vomiting (*p* = 0.022), respectively. The corresponding SB volume was 697 ± 43 and 868 ± 51 mL (*p* = 0.062) (Figure 1b). The mean dose of SB was 30.4 ± 1.6 and 24.5 ± 0.8 Gy in patients with and without early-onset RIE (*p* = 0.002), respectively. The corresponding SB volume was 680 ± 29 and 867 ± 51 mL (*p* = 0.003).

### 3.2. Univariate and Multivariate Analyses of RIE

**Figure 2:**
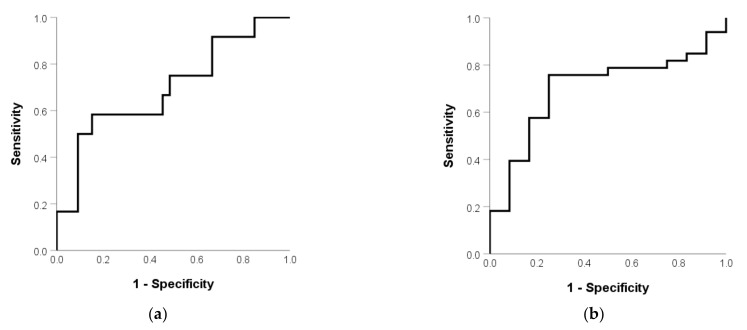
(**a**) Mean SB dose and (**b**) SB volume in patients without and with RIE.

The AUC was 0.697 (*p* = 0.045) (Figure 2a) for predicting vomiting and the optimal cutoff of the SB mean dose was 28.35 Gy. The AUC was 0.699 (*p* = 0.043) (Figure 2b) for predicting vomiting and the optimal cutoff of SB volume was 720 mL. Table 2 shows a univariate analysis of RIE. The incidence of vomiting was 58.3% and 15.2% in patients with a high and low mean SB dose (*p* = 0.007), respectively. The corresponding rate was 52.9% and 10.7% (*p* = 0.004) in patients with a low and high SB volume. The correlation coefficient with SV volume was 0.267 (*p* = 0.076), 0.411 (*p* = 0.005), and -0.146 (*p* = 0.340) in age, BMI, and mean SB dose, respectively. Therefore, BMI was not included in the multivariate analysis because it was not independent with SB volume. Table 3 shows the multivariate analysis of RIE. The mean small-bowel dose (*p* = 0.049) was the independent factor of RIE.

Table 4 shows the multivariate analysis of early-onset RIE. The mean small-bowel dose (*p* = 0.014) remained the independent factor.

## 4. Discussion

Regarding RIE, only sporadic literature is currently discussed [1]. There is no literature on its mechanism in detail or dosimetry. RIE is associated with the location of irradiation [1]. There are some dosimetric studies for RIE in head and neck cancer [4]. Dose to area postrema (AP) and dorsal vagal complex (DVC) may be associated with the development of nausea. It may be involved in the area of the brain stem that is similar to chemotherapy-induced emesis [5]. It is related to serotonin [6]. However, abdominal irradiation is bound to trigger nerve reflexes through the enteric nerve plexus to cause vomiting [5]. Whether it is vomiting caused by chemotherapy or radiotherapy, the clinical treatment medication is the serotonin receptor antagonist [5,7] and the effect is quite good.

In the small intestine, serotonin is distributed in the enterochromaffin cells (ECs) in the epithelial cells, which can be said to be the neuroendocrine cells of the intestinal villi. In fact, serotonin in the SB accounts for about 90% of the whole body [8]. Animal experiments have found that cisplatin induces a vomiting pattern, and serotonin is produced in the proximal part of the small intestine more than in the distal part [5]. Once the EC is stimulated, it can secrete serotonin, which is then used as a neurotransmitter to stimulate the submucosal intrinsic primary afferent neurons and then reflex to the central nervous system [9]. From present results, low-dose radiation may trigger the vomiting reflex if a sufficient volume of SB is irradiated. It is presented as a mean SB dose. The small SB volume possibly associated with vomiting was an unexpected result in present study. No correlation between SB volume and mean SB dose was noted in our analysis. We hypothesize that the vagal tone may affect SB volume. There are some studies discussing the correlation between SB volume and conditions with a decreased vagal tone. Klinge et al. noted a larger SB volume in diabetes patients (mean 927 mL) than health control (mean 713 mL) (*p* = 0.002) [10]. Brock et al. noted a decreased cardiac vagal tone (CVT) in diabetes patients [11]. Age is associated with decreased CVT [12]. In the present data, age and diabetes (data not shown) were associated with an increased SB volume. Therefore, a patient with a small SB volume may have an intense vagal tone that is predisposed to vomiting. Further prospective studies to investigate the correlation between SB volume and vagal tone which is measured by heart rate variability (HRV) [13,14,15] are encouraged.

There are some strengths and limitations in present study. To the best of our knowledge, this is the first study demonstrating the dosimetric correlation of RIE. The mean SB dose was a significant factor. We reported each grade vomiting instead of acute GI toxicity. Although chemotherapy is associated with vomiting, it was not a significant factor in the present study. Acute side effects of CCRT for cervical cancer were usually reported as acute GI toxicities. Vomiting is not separately reported or reported as “nausea or vomiting”, Grade 3–4 is frequently reported instead of each grade. Yang et al. [16] revealed that 70% of patients experienced nausea during EFRT but no vomiting and no dosimetric correlation were reported. Gupta et al. reported 23.3% Grade 3–4 vomiting that is similar to our data during the EFRT [17]. Jakubowicz et al. reported 3.3% of Grade 3–4 nausea and vomiting in patients undergoing a whole-pelvic RT (WPRT) [18]. Uno et al. reported (100%) nausea and vomiting in EFRT patients [19]. Although concurrent chemotherapy is a significant factor of vomiting [20,21], prior chemotherapy is the other significant factor [1,21]. All our patients who did not receive CCRT had experience of prior chemotherapy for endometrial cancer. Therefore, no CCRT patients were chemotherapy naïve in the present study. These can explain no significant role of CCRT in RIE. Ruhlmann et al. proposed that radiation SB volume is a risk factor but no study was available [22]. However, no dosimetric correlation with RIE on small bowel was reported. Therefore, the aim of the current study, is to study this effect. We can set strict constraints of IMRT/VMAT to reduce RIE. In the present patients’ group, the larger upper part of SB irradiated may be a more important factor. Marnitz et al. compared dosimetry among different techniques for WPRT or EFRT and suggested proton beam therapy to reduce complications including SB [23]. In addition, MRI-guided radiotherapy is a possible option to reduce the dose to OARs [24,25]. It may be helpful for reduction in RIE. Early-onset RIE is worth paying attention to because quality of life is always involved. The median onset time of RIE is 3 days after RT starting [1]. In whole body irradiation, prolong emesis lasts 2–3 days in about 40% patients [26]. Delayed emesis as well as cisplatin is not seen with radiotherapy, and anticipatory emesis is extremely rare [26]. Therefore, we defined 72 h as the cutoff time of early-onset RIE. Furthermore, the 10% incidence of emesis (ED10) is 1 Gy [26]. From our data, the mean SB dose of 28.35 Gy at 45 Gy of the prescribed dose is transformed to 1.134 Gy at 1.8 Gy each fraction. The incidence of RIE below 1.134 Gy was 15.2% that is compatible with ED10. The limitation of present study is a retrospective investigation. Statistical power may be influenced by a limited sample size. A further prospective study is needed to clarify why upper abdomen irradiation is a high-risk area for RIE [21,22].

## 5. Conclusions

The SB mean dose is a significant factor of RIE. Constraint of the SB mean dose limited to less than 63% of the 45 Gy prescribed dose is suggested to decrease RIE.

## Figures and Tables

**Table 1:** Characteristics of patients (*n* = 45).

Characteristics	Mean ± SEM or Number (%)
Age (years)	52.0 ± 1.7
BMI (kg/m^2^)	24.5 ± 0.5
Diabetes	
No	40 (88.9%)
Yes	5 (11.1%)
Hypertension	
No	37 (82.2%)
Yes	8 (17.8%)
Disease	
Cervical cancer	37 (82.2%)
Endometrial cancer	8 (17.8%)
Prior chemotherapy	
No	38 (84.4%)
Yes	7 (15.6%)
CCRT	
No	6 (13.3%)
Yes	39 (86.7%)
IMRT/VMAT	
No	13 (28.9%)
Yes	32 (71.1%)
EFRT dose (Gy)	
≤40	6 (13.3%)
45	37 (82.2%)
50–50.4	2 (4.5%)
Small-bowel volume (mL)	
<720	17 (37.8%)
≥720	28 (62.2%)
Small-bowel mean dose (Gy) (%)	
<28.35 (63%)	33 (73.3%)
≥28.35 (63%)	12 (26.7%)

SEM: standard error of mean.

**Table 2:** Univariate analyses of RIE.

Parameters	Category	%	*p* Value
Age (years)	<56	39.3%	0.017
	≥56	5.9%	
BMI (Kg/m^2^)	<24	34.8%	0.208
	≥24	18.2%	
SB volume (mL)	<720	52.9%	0.004
	≥720	10.7%	
Mean SB dose (%)	<63%	15.2%	0.007
	≥63%	58.3%	
IMRT/VMAT	No	7.7%	0.134
	Yes	34.4%	
PTV (mL)	<1635	31.8%	0.445
	≥1635	21.7%	
Prior chemotherapy	No	26.3%	1.000
	Yes	28.6%	
CCRT	No	33.3%	0.650
	Yes	25.6%	

**Table 3:** Multivariate analyses of RIE.

Parameters	Category	OR (95% CI)	*p* Value
SB volume (mL)	<720	reference	0.097
	≥720	0.197 (0.029–1.343)	
Mean SB dose (%)	<63%	reference	0.049
	≥63%	6.104 (1.012–36.8180)	
PTV (mL)	<1635	reference	0.654
	≥1635	1.631 (0.191–13.914)	
Age (years)	<56	reference	0.113
	≥56	0.134 (0.011–1.607)	
IMRT/VMAT	No	reference	0.364
	Yes	3.299 (0.251–43.391)	
CCRT	No	reference	0.646
	Yes	0.525 (0.034–8.196)	

CI: confidence interval; OR: odds ratio.

**Table 4:** Multivariate analyses of early-onset RIE.

Parameters	Category	OR (95% CI)	*p* Value
SB volume (mL)	<720	reference	0.097
	≥720	0.160 (0.018–1.393)	
Mean SB dose (%)	<63%	reference	0.014
	≥63%	13.814 (1.698–112.353)	
PTV (mL)	<1635	reference	0.576
	≥1635	0.527 (0.056–4.982)	
Age (years)	<56	reference	0.118
	≥56	0.112 (0.007–1.740)	
CCRT	No	reference	0.532
	Yes	0.389 (0.020–7.490)	

CI: confidence interval; OR: odds ratio.

## Data Availability

The data presented in this study are available upon request from the corresponding author. The data are not publicly available due to ethical restrictions.

## Abbreviations

Extended-field radiotherapy	EFRT
Whole-pelvic radiotherapy	WPRT
Concurrent chemoradiotherapy	CCRT
Small bowel	SB
Cardiac vagal tone	CVT
Computed tomography	CT
Heart rate variability	HRV
Body mass index	BMI
Intensity-modulated radiotherapy	IMRT
Volumetric modulated arc therapy	VMAT
Planning target volume	PTV

## References

[1] Maranzano E., De Angelis V., Pergolizzi S., Lupattelli M., Frata P., Spagnesi S., Frisio M.L., Mandoliti G., Malinverni G., Trippa F. (2010). A prospective observational trial on emesis in radiotherapy: Analysis of 1020 patients recruited in 45 Italian radiation oncology centres. Radiother. Oncol..

[2] Scarantino C.W., Ornitz R.D., Hoffman L.G., Anderson R.F. (1992). Radiation-induced emesis: Effects of ondansetron. Semin. Oncol..

[3] Roila F., Hesketh P.J., Herrstedt J. (2006). Antiemetic Subcommitte of the Multinational Association of Supportive Care in Cancer. Prevention of chemotherapy- and radiotherapy-induced emesis: Results of the 2004 Perugia International Antiemetic Consensus Conference. Ann. Oncol..

[4] Wang T.J.C., Fontenla S., McCann P., Young R.J., McNamara S., Rao S., Mechalakos J.G., Lee N.Y. (2013). Correlation of planned dose to area postrema and dorsal vagal complex with clinical symptoms of nausea and vomiting in oropharyngeal cancer (OPC) patients treated with radiation alone using IMRT. J. Radiat. Oncol..

[5] Endo T., Minami M., Hirafuji M., Ogawa T., Akita K., Nemoto M., Saito H., Yoshioka M., Parvez S.H. (2000). Neurochemistry and neuropharmacology of emesis—The role of serotonin. Toxicology.

[6] Naylor R.J., Rudd J.A. (1996). Mechanisms of chemotherapy/radioterapy-induced emesis in animal models. Oncology..

[7] Smith H.S., Cox L.R., Smith E.J. (2012). 5-HT3 receptor antagonists for the treatment of nausea/vomiting. Ann. Palliat. Med..

[8] Bertrand P.P., Bertrand R.L. (2010). Serotonin release and uptake in the gastrointestinal tract. Auton. Neurosci..

[9] Gershon M.D. (2004). Review article: Serotonin receptors and transporters—Roles in normal and abnormal gastrointestinal motility. Aliment. Pharmacol. Ther..

[10] Klinge M.W., Sutter N., Mark E.B., Haase A.M., Borghammer P., Schlageter V., Lund S., Fleischer J., Knudsen K., Drewes A.M. (2021). Gastric emptying time and volume of the small intestine as objective markers in patients with symptoms of diabetic enteropathy. J. Neurogastroenterol. Motil..

[11] Brock C., Jessen N., Brock B., Jakobsen P.E., Hansen T.K., Rantanen J.M., Riahi S., Dimitrova Y.K., Dons-Jensen A., Aziz Q. (2017). Cardiac vagal tone, a non-invasive measure of parasympathetic tone, is a clinically relevant tool in Type 1 diabetes mellitus. Diabet. Med..

[12] Farmer A.D., Coen S.J., Kano M., Weltens N., Ly H.G., Botha C., Paine P.A., Oudenhove L.V., Aziz Q. (2014). Normal values and reproducibility of the real-time index of vagal tone in healthy humans: A multi-center study. Ann. Gastroenterol..

[13] Thompson J.J., Elsenbruch S., Harnish M.J., Orr W.C. (2002). Autonomic functioning during REM sleep differentiates IBS symptom subgroups. Am. J. Gastroenterol..

[14] Liu Q., Wang E.M., Yan X.J., Chen S.L. (2013). Autonomic functioning in irritable bowel syndrome measured by heart rate variability: A meta-analysis. J. Dig. Dis..

[15] Pellissier S., Dantzer C., Mondillon L., Trocme C., Gauchez A.S., Ducros V., Mathieu N., Toussaint B., Fournier A., Canini F. (2014). Relationship between vagal tone, cortisol, TNF-alpha, epinephrine and negative affects in Crohn’s disease and irritable bowel syndrome. PLoS ONE.

[16] Yang B., Liu X., Hu K., Qiu J., Zhang F., Hou X., Yan J., Meng Q., Wang W., Yu L. (2019). Reduction of dose to duodenum with a refined delineation method of para-aortic region in patients with locally advanced cervical cancer receiving prophylactic extended-field radiotherapy. Radiat. Oncol..

[17] Gupta M., Chopra S., Kunder S., Dheera A., Sampathirao D., Engineer R., Ghosh J., Gurram L., Mahantshetty U., Gupta S. (2019). Early toxicity and treatment outcomes of extended field-intensity modulated radiotherapy for cervical cancer patients with para-aortic nodal metastasis. Ecancermedicalscience.

[18] Jakubowicz J., Blecharz P., Skotnicki P., Reinfuss M., Walasek T., Luczynska E. (2014). Early toxicity and treatment outcomes of extended field-intensity modulated radiotherapy for cervical cancer patients with para-aortic nodal metastasis. Eur. J. Gynaecol. Oncol..

[19] Uno T., Mitsuhashi A., Isobe K., Yamamoto S., Kawakami H., Ueno N., Usui H., Tate S., Kawata T., Ito H. (2008). Concurrent daily cisplatin and extended-field radiation therapy for carcinoma of the cervix. Int. J. Gynecol. Cancer.

[20] Peters W.A., Liu P.Y., Barrett R.J., Stock R.J., Monk B.J., Berek J.S., Souhami L., Grigsby P., Gordon W., Alberts D.S. (2000). Concurrent chemotherapy and pelvic radiation therapy compared with pelvic radiation therapy alone as adjuvant therapy after radical surgery in high-risk early-stage cancer of the cervix. J. Clin. Oncol..

[21] McKenzie E., Zaki P., Raman S., Olson R., McFarlane T., DeAngelis C., Chan S., Pidduck W., Razvi Y., Bushehri A. (2019). Radiation-induced nausea and vomiting: A comparison between MASCC/ESMO, ASCO, and NCCN antiemetic guidelines. Support. Care Cancer.

[22] Ruhlmann C.H., Jahn F., Jordan K., Dennis K., Maranzano E., Molassiotis A., Roila F., Feyer P. (2017). 2016 updated MASCC/ESMO consensus recommendations: Prevention of radiotherapy-induced nausea and vomiting. Support. Care Cancer.

[23] Marnitz S., Wlodarczyk W., Neumann O., Koehler C., Weihrauch M., Budach V., Cozzi L. (2015). Which technique for radiation is most beneficial for patients with locally advanced cervical cancer? Intensity modulated proton therapy versus intensity modulated photon treatment, helical tomotherapy and volumetric arc therapy for primary radiation—An intraindividual comparison. Radiat. Oncol..

[24] Massaccesi M., Cusumano D., Boldrini L., Dinapoli N., Fionda B., Teodoli S., Azario L., Mattiucci G.C., Balducci M., Cellini F. (2019). A new frontier of image guidance: Organs at risk avoidance with MRI-guided respiratory-gated intensity modulated radiotherapy: Technical note and report of a case. J. Appl. Clin. Med. Phys..

[25] Boldrini L., Piras A., Chiloiro G., Autorino R., Cellini F., Cusumano D., Fionda B., D’Aviero A., Campitelli M., Marazzi F. (2020). Low Tesla magnetic resonance guided radiotherapy for locally advanced cervical cancer: First clinical experience. Tumori.

[26] Feyer P.C., Stewart A.L., Titlbach O.J. (1998). Aetiology and prevention of emesis induced by radiotherapy. Support. Care Cancer.

